# Targeting Heat Shock Protein 27 in Cancer: A Druggable Target for Cancer Treatment?

**DOI:** 10.3390/cancers11081195

**Published:** 2019-08-16

**Authors:** Seul-Ki Choi, Heejin Kam, Kye-Young Kim, Suk In Park, Yun-Sil Lee

**Affiliations:** Graduate School of Pharmaceutical Sciences, College of Pharmacy, Ewha Womans University, Seoul 03760, Korea

**Keywords:** heat shock protein 27, HSP27 inhibitor, anti-cancer drugs, resistance

## Abstract

Heat shock protein 27 (HSP27), induced by heat shock, environmental, and pathophysiological stressors, is a multi-functional protein that acts as a protein chaperone and an antioxidant. HSP27 plays a significant role in the inhibition of apoptosis and actin cytoskeletal remodeling. HSP27 is upregulated in many cancers and is associated with a poor prognosis, as well as treatment resistance, whereby cells are protected from therapeutic agents that normally induce apoptosis. This review highlights the most recent findings and role of HSP27 in cancer, as well as the strategies for using HSP27 inhibitors for therapeutic purposes.

## 1. Introduction

The heat shock protein (HSP) is a protein family produced in cells by stressors, such as hypoxia, hyperoxia, UV light exposure, viral agents, and nutritional deficiencies. Its primary role is to maintain cellular homeostasis, promoting cell survival in lethal conditions. It associates with major regulatory proteins, such as transcriptional factors, protein kinases, and hormone receptors [1]. HSPs protect cells by acting as molecular chaperones which correct misfolded proteins. There are many types of HSPs, and their functions vary slightly. Although there are a few ways to classify HSPs, one way is to categorize them by their molecular weight. For example, the HSP27 molecule is 27 kDa in size. Using their molecular weights, mammalian HSPs can be classified into six families: HSP100, HSP90, HSP70, HSP60, HSP40, and small HSPs (sHSP, 15 to 30 kDa) [2].

**Figure 1:**
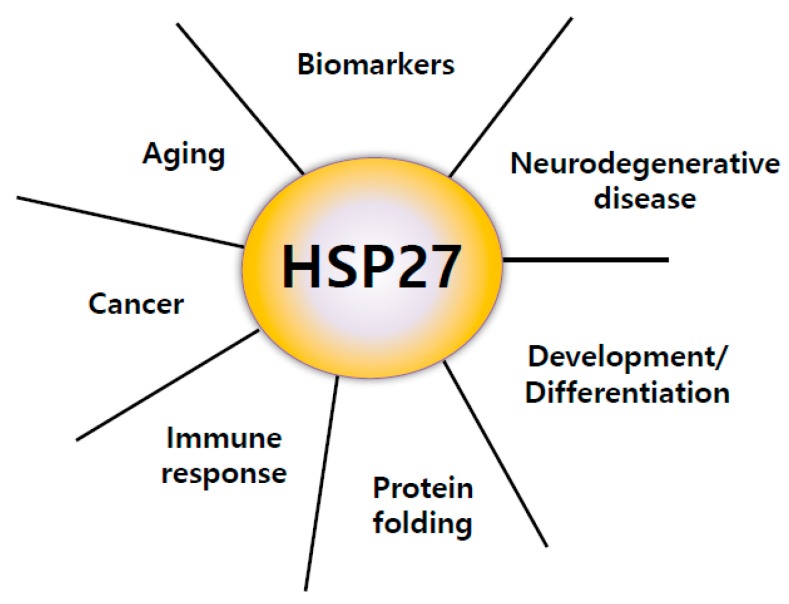
Major roles of heat shock protein 27 (HSP27). HSP27 has important functions, including protein folding regulation by chaperone activity, immune response, cancer promotion, inducing resistance to anticancer drugs, aging, biomarkers of several diseases, aggravation of neurodegenerative disease, development, and differentiation [5,6,7,8].

HSP27 is a type of small Heat Shock Protein (sHSP). The mammalian sHSP family, also known as the HSPB family, contains ten members: HSPB1 to HSPB10, HSP27/HSPB1, MKBP (Myotonic dystrophy protein kinase-binding protein)/HSPB2, HSPB3, αA-crystallin/HSPB4, αB-crystallin/HSPB5, HSP20/HSPB6, cvHSP (cardiovascular heat shock protein)/HSPB7, HSP22/HSPB8, HSPB9, and ODF1 (outer dense fiber protein)/HSPB10 [3]. HSP27 is encoded on the *HSPB1* gene and belongs to a family of ATP-independent chaperones. HSP27 is a single copy gene covering 2.2 kb transcripts organized into three exons encoding a 205 amino acid protein. The mechanism of its substrates and chaperone function have not been fully studied compared to other large HSPs. HSP27 is reported to be involved in cell resistance to stress factors and heat shock. However, the function of HSP27 is hijacked during disease, and HSP27 helps to promote disease, rather than appropriately regulate cell homeostasis. HSP27 is present in both the cytoplasm and nucleus. Heat shock and exposure to various stressful conditions can cause it to localize to the nucleus. It was shown that the overexpression of HSP27 promoted recovery from the aggregation of heat-induced nuclear-protein [4], suggesting that HSP27 was partly responsible for subsequent cell survival. Therefore, HSP27 plays a fundamental role in cell physiology in various disease states, including cancer (Figure 1).

In the following section, we present an overview of HSP27 and discuss the highly complex patterns of HSP27 phosphorylation and oligomerization related to its function. We also examine inhibitors targeted to HSP27 as cancer treatment strategies.

## 2. Structure of HSP27

Small HSPs are the most diverse in structure among the molecular chaperones. HSP27 contains a highly conserved α-crystallin domain. [4]. In humans, the α-crystallin domain plays a crucial role in dimer formation [9]. The structure of α-crystallin is dynamic and is affected by rapid subunit exchange under stressful conditions [10]. HSP27 acts as a chaperone to form multimeric complexes in cells and to stabilize denatured or aggregated proteins and return them to their original form [11]. Since the oligomeric conformation of HSP27 and the monomeric form occur dynamically, further research is needed to determine whether oligomers or monomers are required for protein homeostasis and what they do.

X-ray analysis has revealed the crystallin domain of HSP27; however, the entire molecular structure is still unknown because it was difficult to obtain a stable crystal of HSP27’s oligomeric protein [12]. Therefore, by using Psipred [13], a secondary structure helix and strand was deduced from the amino acid sequence of HSP27. A complete tertiary structure has not yet been obtained and may not be obtainable due to the coil being too flexible. HSP27 has a poorly conserved, disorganized N-terminal and a highly flexible, variable C-terminal. HSP27 is likely to change its structure depending on the conditions, such as the pH and temperature [14]. It might be possible to obtain a tertiary structure under very specific conditions. HSP27 contains a poorly-conserved WDPF domain region, a highly-conserved α-crystallin domain region with β-sheets, a partially conserved PSRLFDQXFGEXLL sequence, and a flexible C-terminal. The WDPF domain name was derived from the amino acid residues it contains, including W (tryptophan), D (aspartic acid), P (proline), and F (phenylalanine). The α-crystallin structure is important for oligomerization and solubility [15]. HSP27 is an ATP-independent molecular chaperone involved in protein folding-refolding machinery [16]. Several studies have shown that constitutively expressed HSP27 has irrelevant cellular functions that can lead to interact with many other protein partners [17]. Therefore, it is crucial to understand the structure of HSP27 to grasp the structure–function relationship, including modulation of the activity and half-lives of many crucial client polypeptides [18]. One of the major challenges in HSP27 research is determining the factors that affect its activity level, such as the extent of oligomerization, the interaction with protein partners, etc., which may lead to the development of potential anticancer therapeutics by modulating HSP27 activity. It is logical to assume that both the oligomerization levels and the interaction with protein partners are involved, but this is very difficult to determine with certainty because the ratio of HSP27 polypeptides interacting with partners can be highly variable [19].

## 3. Oligomerization and Phosphorylation of HSP27

HSP27 is phosphorylated in response to a variety of stressors. On the molecular level, HSP27 is phosphorylated by various protein kinases, which in turn can be controlled by various factors such as tumor necrosis factor-α (TNF-α); interleukin-1β (IL-1 β); transforming growth factor-beta (TGF-β); mitogens, such as insulin-like growth factor-1 (IGF-1); and steroid hormones [20]. HSP27 can form oligomers up to 1000 kDa. α-crystallin plays a vital role in oligomerization as it forms a dimer, the molecular base of the oligomeric complex. Moreover, a conserved tripeptide (I/V/L)-X-(I/V/L) motif on the C-terminal interacts with a hydrophobic groove on the surface of the core α-crystallin domain of a neighboring dimer. The dimer of HSP27 acts as a building block for multimeric complexes. Therefore, it can control the structural plasticity of oligomeric sHSP [21].

The oligomerization of HSP27 is regulated by phosphorylation. When HSP27 is not phosphorylated, it forms an oligomer. Electronic microscopy and X-ray crystallography images indicate that oligomers form ring-like structures with symmetrically packed dimers inside [22]. Since αB-crystallin subunits cannot interact with unfolded proteins, phosphorylated HSP27 demonstrates decreased chaperone activity. Therefore, oligomerization and phosphorylation direct the biological activity and function of HSP27 [14,23,24].

**Figure 2:**
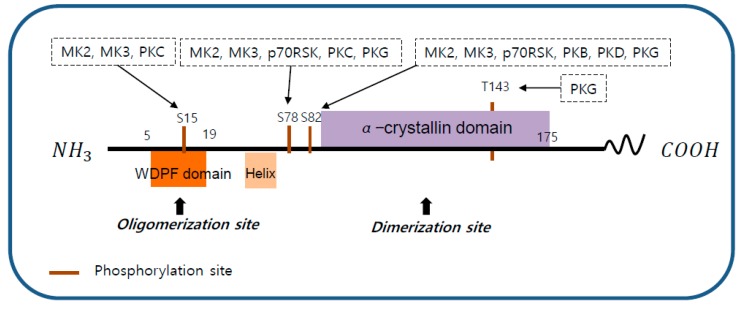
The structure of heat shock protein 27 (HSP27). The structure of human HSP27 consists of the N-terminal domain, the α-crystallin domain, and the C-terminal domain. The N-terminal domain contains a WDPF motif which is essential for large oligomerization. The C-terminal domain includes an α-crystallin motif that is highly conserved between species and is involved in the formation of small oligomerization. HSP27 phosphorylation sites S15, S78, S82, and T143 are indicated. S15 can be phosphorylated by p38 mitogen-activated protein kinase (MAPK)-activated protein kinase 2 (MK2) and 3 (MK3), and protein kinase C (PKC). S78 can be phosphorylated by MK2, MK3, ribosomal S6 kinase (p70RSK), PKC, and protein kinase G (PKG). S82 can be phosphorylated by MK2, MK3, p70RSK, protein kinase B (PKB), protein kinase D (PKD), and PKG. T143 can be phosphorylated by PKG.

Human HSP27 can be phosphorylated on three serine residues (S15, S78, and S82) and threonine (T143) by multiple kinases, including the p38 mitogen-activated protein kinase (p38 MAPK) pathway and ribosomal S6 kinase (p70RSK), protein kinase B (PKB), protein kinase C (PKC), protein kinase D (PKD), and protein kinase G (PKG) [25,26,27] (Figure 2).

**Figure 3:**
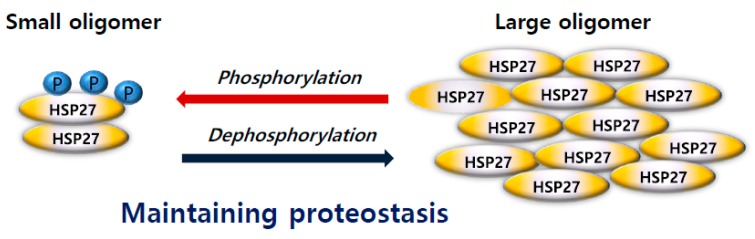
Phosphorylation induced conformational structural switching between different states. Heat shock protein 27 (HSP27) exists as large oligomers when unphosphorylated. At specific serine residues in the mitogen-activated protein kinase (MAPK) pathway, HSP27 switches to smaller oligomers. HSP27 conformational structure changes actively and contributes to maintaining proteostasis.

Previous studies have reported that S78 and S82 significantly contribute to the oligomerization of HSP27, but that S15 has only minor effects. [25,26]. Phosphorylation promotes the formation of small oligomers, while dephosphorylation promotes the formation of large oligomers and is a reversible event that regulates protein oligomerization [25,26]. HSP27 can form oligomers up to 1000 kDa, which is a very dynamic process that plays a central role in modulating the chaperone activity of HSP27, a competent, binding state of the client protein [28] (Figure 3). According to recent studies, the dimeric form of HSP27 is central to its enhanced chaperone activity, demonstrated by increased binding to other client proteins [29,30]. Immediate and transient phosphorylation of HSP27 is reported to initiate chemoresistance in cancer cells [31]. 

The α-crystallin domain of murine (C141) and human (C137) HSP27 [17] and that on the beta-7 strand of several human other HSPs has been proposed to play a pivotal role in the inter-subunit contact of several human sHSPs [32]. Deletion or mutation of the unique cysteine blocks dimer formation, which consequently alters multimer formation, suggesting that the cysteine residue of HSP27 is important for its chaperone activity and its ability to interact with many polypeptides [33]. 

## 4. The Role of HSP27 in Cancer

**Figure 4:**
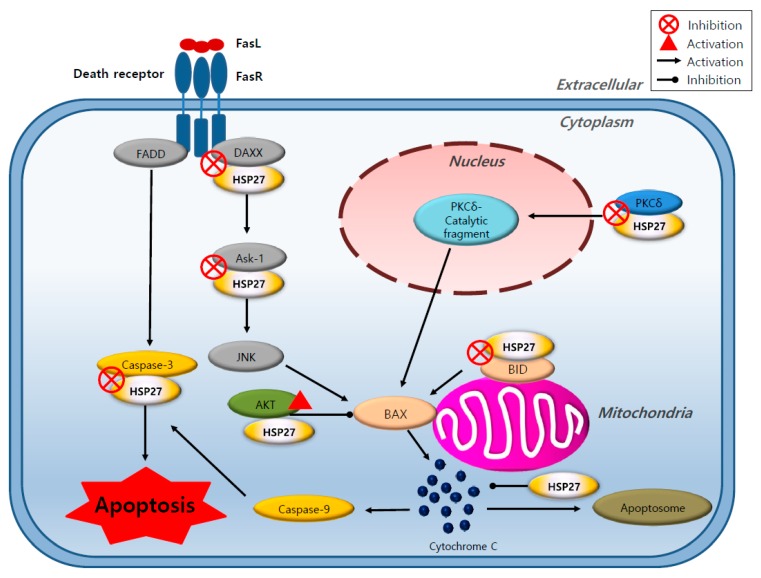
Role of heat shock protein 27 (HSP27) in different cellular apoptotic processes. HSP27 inhibits apoptosis by integrating with different signaling pathways, including the extrinsic and intrinsic apoptosis pathway. HSP27 inhibits Bcl-2-associated X protein (BAX) by directly binding to death domain associated protein (DAXX) or apoptosis signal-regulating kinase-1 (Ask-1) to inhibit its function, which enhances AKT activity inhibiting BH3 interacting-domain death agonist (BID) or protein kinase C delta type (PKC δ) function. HSP27 inhibits caspase 3, which directly functions in cellular apoptosis. HSP27 contributes to cell survival.

Overexpression of HSP27 is closely related to tumorigenesis, metastasis, and invasiveness, and thus, to poor prognosis in various cancers [34,35]. An increased expression of HSP27 is also found to be associated with resistance to chemotherapy drugs in cancer cells [36]. The cytoprotective function of HSP27 is associated with chaperone functions, direct interference with the apoptosis pathway, the promotion of drug resistance, and the regulation of cytoskeleton dynamics [37]. HSP27 has been shown to protect cells from death signals induced in different ways, including apoptosis, necrosis, and various physiological stresses [38,39]. HSP27 inhibits both intrinsic and extrinsic apoptotic pathways through binding of its small or large oligomeric form to cytochrome C or death domain associated protein (DAXX), respectively [40,41]. HSP27 inhibits caspase 9, depending on the activity of Bcl-2-associated X protein (BAX), which is activated by BH3 interacting-domain death agonist (BID). HSP27 also interacts with protein kinase C delta type (PKC δ) and induces resistance to cancer therapy [42]. Moreover, the interaction between HSP27 and nuclear factor of kappa light polypeptide gene enhancer in B-cells inhibitor, alpha (IkBα) is involved in the activation of the nuclear factor kappa-light-chain-enhancer of activated B cells (NFkB) [43]. It can interact with the microtubule actin protein, which is imperative for maintaining cytoskeleton integrity and may help to promote cell survival and invasion [44] (Figure 4).

Antisense oligonucleotides and small interfering RNA (siRNA) to HSP27 increase apoptotic rates and enhance chemotherapy activity [1]. HSP27 is highly expressed in anti-cancer drug-resistant cancers. Studies have reported increased levels of HSP27 in various types of cancer, such as that of the liver, breast, colorectal, melanoma, prostate, glioma, lung, gastric, rectal, pancreatic, and kidney (Table 1). Therefore, HSP27 can be an important therapeutic target, especially in cancer, because it plays a significant role in cell apoptosis or multiple cellular pathways under stress conditions in cells.

**Figure 5:**
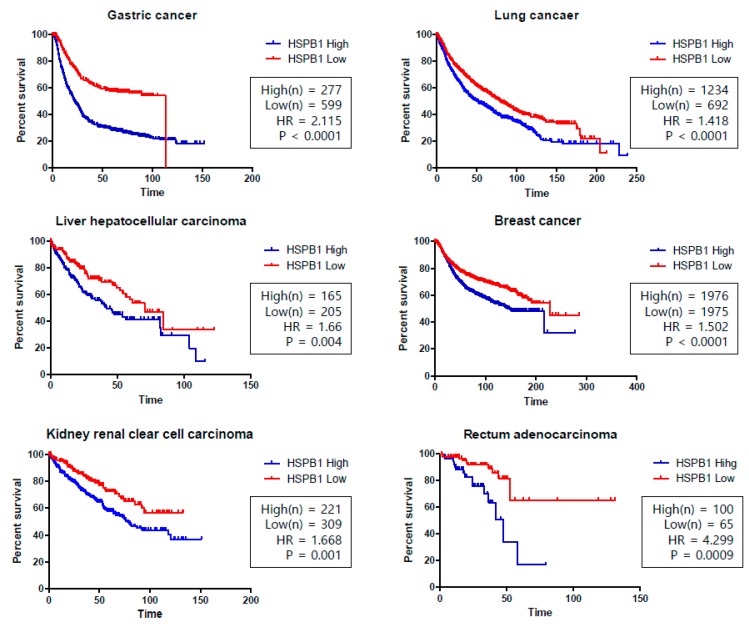
Kaplan–Meier (KM) curves for *HSPB1* (gene name of heat shock protein 27 (HSP27)) in the overall survival of various cancers. Gastric cancer, lung cancer, hepatocellular carcinoma, breast cancer, clear cell renal carcinoma, and rectum adenocarcinoma show high survival rates associated with low HSPB1 expression. *p*-values were calculated using the log-rank test. The Hazard Ratio (HR) is the ratio of the hazard rates corresponding to the conditions described by two levels of an explanatory variable (HR > 1 was considered a higher hazard of death from the *HSPB1*-High group). An independent univariate survival analysis of overall survival (OS) was analyzed based on a merged data set from the Kaplan–Meier Plotter [76]. Data were derived from http://kmplot.com/analysis/ and survival curves were drawn using PRISM software [77].

In cancer patients, the overexpression of HSP27 is associated with a poor prognosis and HSP27 has become the focus of research investigating factors involved in the invasiveness and metastasis affecting key determinants for overall survival. Recent studies analyzed the HSP27 levels in serum and tumor microenvironments, and the serum HSP27 levels were significantly higher in patients with prostate and breast cancer than in the control group [72,73,74,75]. Moreover, HSP27 levels were also reported to be related to the overall survival of patients with many other types of cancer, such as gastric, lung, liver, breast, kidney, and rectum adenocarcinoma (Figure 5).

Recent clinical trials have investigated the inhibition of HSP27 as a molecular target for cancer therapy. However, unlike other HSPs, which bind ATP, HSP27 is an ATP-independent chaperone, and this makes targeting HSP27 difficult with small compounds [78]. However, a recent study suggested a novel strategy to inhibit HSP27 by inducing the cross-linking of HSP27 proteins [79]. By inserting between the disulfide bond of HSP27, the cross-linking of HSP27 was altered, and the normal HSP27 dimerization was disrupted, which resulted in the inhibition of functional HSP27.

**Figure 6:**
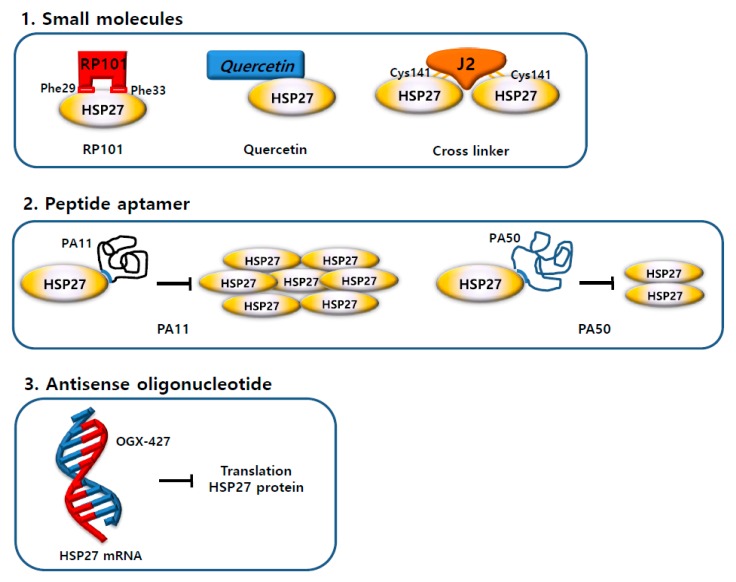
Strategies of heat shock protein 27 (HSP27) inhibitors. (**1**) Three small molecule inhibitors (Brivudine (RP101), Quercetin, and cross-linker) bind directly to the HSP27 protein and inhibit the activity of the HSP27 protein. (**2**) Peptide aptamers (PA11 and PA50) bind directly to the HSP27 protein and inhibit oligomerization or dimerization. (**3**) Antisense oligonucleotide (OGX-427) binds to HSP27 mRNA and prevents translation of the HSP27 protein. As a result, the amount of HSP27 protein is reduced.

Moreover, the altered dimerization of HSP27 can sensitize cancer cells with a high HSP27 expression [63]. This strategy is expected to overcome drug development currently limited by the absence of HSP27 inhibitors and presents the possibility of the development of novel HSP27 inhibitors (Figure 6).

## 5. HSP27 Inhibitors for Cancer Treatment

### 5.1. Small Molecules

#### 5.1.1. RP101 (Brivudine)

RP101 (known as BVDU, bromovinyldeoxyuridine, brivudine) is a nucleoside that can inhibit HSP27 function via binding π-stacking with Phe29 and Phe33 of HSP27. When RP101 binds to HSP27, the binding of HSP27 to Akt1, pro-caspase3, and cytochrome C is weakened, which affects apoptosis [80,81]. Therefore, RP101 functions as a chemo-sensitizing agent to anti-cancer drugs. In vitro experiments showed that mitomycin C (MMC) with RP101 inhibited cell growth after heat shock [82]. RP101 inhibited the resistance of rat sarcoma cells to MMC by reducing their growth by 5-fold compared to the MMC alone group [80]. RP101 combined with gemcitabine in fibrosarcoma cells reduced invasiveness by 30–50% compared to gemcitabine alone. RP101 with either cisplatin or cyclophosphamide significantly inhibited the tumor growth of AH13r sarcoma-grafted SD-rats [80]. These data suggest that RP101 combination is more effective than cytotoxic drug monotherapy. In clinical studies, RP101 increased the overall survival rate of patients with pancreatic cancer by 8.5 months compared with the control group [80]. Phase II clinical trials for the treatment of pancreatic cancer using gemcitabine with RP101 increased the median survival by approximately 2.17 months. However, overuse of RP101 showed increased toxic side effects from gemcitabine in some patients [80], indicating the limitation of RP101 in clinical application (Table 2).

#### 5.1.2. Quercetin

Quercetin, a bioflavonoid widely distributed in plants, is a well-known natural compound with anti-cancer properties [83]. Quercetin suppresses the heat shock transcriptional factor1 (HSF1) dependent induction of the HSPs [84,85] and demonstrations anti-tumor effects in oral, hepatoma, prostate cancer, glioblastoma, squamous, gastric, and breast cell lines, and various cancer stem cells [86,87,88,89,90,91]. In lung cancer cells (A549), cisplatin or gemcitabine against A549 cells inhibits cell viability with quercetin compared to alone [92]. Quercetin acts as a chemo-sensitizer when used with first-line chemotherapeutic drugs such as 5-fluorouracil, gemcitabine, doxorubicin, and cisplatin. However, quercetin’s exact mechanism has not been identified, and additional research is needed to inhibit HSP27 directly. A recent study suggested that quercetin directly inhibits the cellular expression of casein kinase 2 (CK2) [93]. Moreover, the knock down of CK2 promotes the proteasomal degradation of HSP27, suggesting that CK2 directly increases the stability of HSP27 [94], indicating a possible role of quercetin as a regulator of HSP27 protein stability by inhibiting CK2 (Table 2). 

#### 5.1.3. Altered Dimerization of HSP27 Using Small Molecules

**Figure 7:**
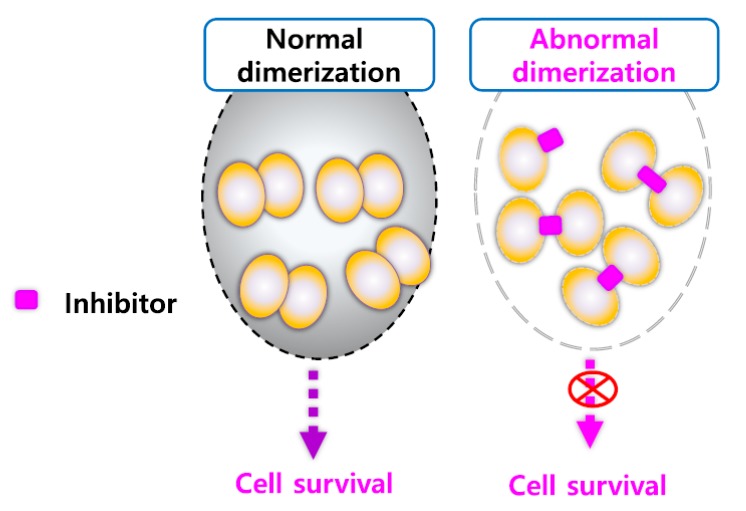
Altered cross-linking of heat shock protein 27 (HSP27) using small molecules for HSP27 inhibition. Scheme for the mechanism of HSP27-cross-linking by small molecules. Normal dimerization of HSP27 contributes to cancer cell survival, but abnormal dimerization of HSP27 using small molecules causes cancer cell death.

According to recent studies, zerumbone [79], isolated from a natural product, and SW15, a synthetic xanthone compound, induced cross-linking of the HSP27 protein. It forms a covalent bond between the cysteine—thiol group of HSP27 and forms an abnormal dimerization [95]. The same xanthone moiety with different side chains caused a different type of HSP27 cross-linking activity. The HSP27 Cys residue is necessary for the altered cross-linking of HSP27 by the xanthone compound [63]. The combination of anti-cancer drugs and the xanthone compound sensitized NSCLC cells. The Cys residue of HSP27 is vital for the sensitization of cancer cells by the xanthone compound in combination with anti-cancer drugs. Additionally, the xanthone compound sensitized cancer cells in combination with radiation. J2, a synthetic chromone compound, has a pharmacophore structure and more potent cross-linking activity than SW15 [63]. Therefore, the alteration of cross-linking is considered to be a novel strategy for the inhibition of HSP27-mediated resistance in lung cancer (Figure 7, Table 2).

### 5.2. Antisense Drug

A second-generation antisense oligonucleotide (ASO) targeting HSP27 mRNA, OGX-427 (OncoGeneX Pharmaceuticals, Bothell, Washington, USA), decreases the expression of HSP27. In a prostate cancer xenograft, OGX-427 with chloroquine reduces the tumor volume compared to chloroquine alone [96]. Pancreatic and lung cancer xenografts also showed that when combined with gemcitabine, erlotinib and OGX-427 decrease tumor size compared to alone [96,97,98]. In phase I of the clinical study, the response occurred in 33% of the 15 metastatic bladder cancer patients. In phase II on castrate-resistant prostate cancer patients, 71% of patients presented as progression-free at 12 weeks when OGX-427 was combined with prednisone [99].

Inhibition of HSP27 significantly increased radiation-induced apoptosis and clonogenic death and promoted Akt inactivation. HSP27 knockdown improved the efficacy of radiation therapy by enhancing the cytotoxic effects of radiation therapy in patients with radiation-resistant lung cancers [100]. The combination of OGX-427 and local tumor irradiation resulted in significant regression in SQ20B cancer cell-bearing mice and a decrease in glutathione antioxidant defenses and cell survival [100].

Tumor therapy with radiation to OGX-427 resulted in reduced angiogenesis associated with decreased activation of the Akt pathway. The combination therapy improved the survival and anti-cancer effect of Sq20B cancer cell-bearing mice and did not show signs of acute or delayed toxicity [100]. However, a clinical study reported that the addition of OGX-427 treatment to a standard chemotherapy regimen did not result in increased survival in unselected patients with metastatic pancreatic cancer, but there was a trend toward prolonged progression-free survival and overall survival in patients with high baseline serum HSP27, suggesting that this therapy may warrant further evaluation in this subgroup [101].

### 5.3. Peptide Aptamers

The use of specific peptides to inhibit the anti-apoptotic activity of HSP27 has become a new approach to chemotherapy because of the difficulty of dealing with antisense technology in vivo. Protein aptamers are small amino acid sequences that are inserted into a scaffold protein. They bind to specific protein domains and are designed to regulate the activity of various cellular proteins, including oncogenes, transcription factors, cell cycle regulators, and others [102]. Recent research showed that peptide aptamers could interact with HSP27 and promote the apoptosis of cancer cells. PA11 and PA50 specifically bind to HSP27, interfering with the dimerization and oligomerization of HSP27, and could act as negative regulators of HSP27 functions. PA11 prevents HSP27 oligomerization, which ultimately results in the inability of HSP27 to inhibit cellular proteostasis. PA50 primarily inhibits HSP27 dimerization, disrupting the essential processes of cell survival by destroying the ability of HSP27 to participate in cell signaling events. These peptide aptamers also showed anti-tumor effects in mouse xenograft models [102]. Similar to the small molecule inhibitors of HSP27, a peptide aptamer has a powerful effect when used with other anti-cancer drugs more than when used alone. 

Even though the pre-clinical success of peptide aptamers suggests a potential application to cancer therapy, there are limitations to the use of protein aptamers, including restrictions with the size of the investigated protein, and the inability to deal with protein complexes and membrane components, as well as the difficulties of working in an RNase-free environment. Once these limitations are overcome, protein aptamers can be used to specifically target HSP27 to explore new insights into the HSP27 structure–function relationship and discover novel anticancer drugs.

## 6. Conclusions

In this review, we have discussed the role of HSP27 under stress conditions, particularly cancer, with regard to the interaction of small heat shock proteins with other cellular molecules. The HSP27 is one of the cellular regulatory factors that aid in maintaining proteostasis and undergoes phosphorylation at multiple serine residues, which is thought to regulate its function. However, there has been no consensus as to the overall effect of phosphorylation. The structure of HSP27 differs greatly, depending on the species, but functional forms such as oligomeric and dimeric forms of HSP27 are essential. HSP27 is crucial in the regulation of the development, progression, and metastasis of cancer, as well as in cell apoptosis and drug resistance, and it may be an indicator of poor disease prognosis. HSP27 is overexpressed in a variety of cancers and can be used as a biomarker in cancer diagnosis and prognosis. Therefore, there is a need for research on diseases related to HSP27 and the development of drugs in the future. HSP27 also modulates drug resistance and is a potential target for a chemotherapeutic agent. Therefore, the structural complexity of HSP27 challenges the discovery of therapeutic inhibitors that can neutralize HSP27 functionality. Moreover, recent studies have suggested the potential of inhibiting HSP27 as a therapeutic target for cancer. However, it is believed that, unlike other heat shock proteins such as HSP70 and HSP90, the sHSPs, such as HSP27, lack an ATP binding site and this makes it difficult to think of HSP27 as an easy target for a small molecule. Since antisense oligonucleotide drugs such as OGX-427 are still undergoing clinical trials, researchers should focus their efforts in this direction to investigate potential new cancer therapies. In this sense, small molecules of cross-linking HSP27 may be promising for functionally inhibiting HSP27.

In conclusion, HSP27 is frequently overexpressed in many cancers and is associated with the development of resistance against anti-cancer drugs. Therefore, inhibitors of HSP27 may improve cancer chemotherapy when used as combination-therapy together with anti-cancer drugs.

## Figures and Tables

**Table 1:** Functions of heat shock protein 27 (HSP27) in various cancer cells (year 2010~).

Cancers	Effects	References
Liver	Promotes proliferation and invasion of hepatocellular carcinoma cells.	[45,46]
Breast	Downregulation of HSP27 induces chemo-sensitization to Herceptin and inhibition of cancer cell proliferation. HSP27 regulates the EMT process and NFkB activity to contribute to the maintenance of BCSCs. Expression of phosphorylated forms of the chaperone *HSPB1* correlates with the amount and percentage of lymph node metastases. Down-regulation of HSP27 in human breast cancer cells modulates down-regulation of PTEN.	[47,48,49,50]
Colorectal	Suppression of HSP27 protein expression enhances 5-FU sensitivity. Patients with low HSP27 expression show better survival than those with high HSP27 expression. Acquired drug resistance of 5-FU is caused by the enhanced constitutive expression of HSPB1 and its phosphorylated form in colorectal cancer cells.	[51,52,53]
Melanoma	HSP27 expression is associated with impaired prognosis in melanoma. HSP27 is important for tumor dormancy, angiogenesis regulation, and tumor progress in cutaneous melanoma.	[54,55]
Prostate	HSP27 increases PCa cell motility, growth, and survival. Downregulation of HSP27 radiosensitizes human prostate cancer cells. In patients with prostate cancer, with HSP27 and Twist expression, each is elevated in high-grade prostate cancer tumors. DNA methylation of *HSPB1* resulted in a poor outcome in prostate cancer patients.	[35,56,57,58]
Glioma	Promotes glioma cell proliferation. Quantitative proteomic analysis shows that HSP27 is involved in the poor prognosis of GBN.	[59,60]
Lung	HSP27 inhibitor induces chemo-sensitization to anti-cancer drugs. Increased HSP27 expression correlates with shorter survival of NSCLC patients.	[61,62,63]
Gastric	Meta-analysis of gastric cancer is strongly dependent on the overexpression of HSP27.	[64,65]
Rectal	High expression of HSP27 represents poor survival in rectal cancer.	[66]
Pancreatic	Downregulation of HSP27 sensitizes to gemcitabine in the gastric cancer cell line by regulating the expression of Snail. HSP27 phosphorylation status contributes to gemcitabine resistance.	[67,68]
Kidney	Abnormal HSP27 phosphorylation is observed in renal cancers, as well as in other kidney diseases. In ccRCC patients, high serum HSP27 is associated with high-grade (Grade 3–4) tumors. TGF-β1/p38/HSP27 signaling pathway inhibits cancer invasion and metastasis in RCC.	[69,70,71]

PTEN, phosphatase and tensin homolog; EMT, epithelial-mesenchymal transition; BCSC, breast cancer stem cell; NFkB, nuclear factor kappa-light-chain-enhancer of activated B cells; 5-FU, fluorouracil; PCa, prostate cancer; NSCLC, non-small cell lung cancer; GBN, glioblastoma; ccRCC, clear cell renal cell carcinoma; RCC, renal cell carcinoma; TGF-β1, transforming growth factor beta.

**Table 2:** Structure and mechanism of action of small-molecule heat shock protein 27 (HSP27) inhibitors.

Name	Structure	Mechanism
RP101	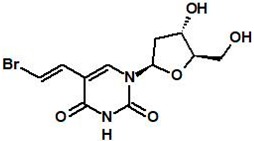	Inhibits HSP27 protein by π-stacking binding to Phe29 and Phe33 of HSP27 [80].
Quercetin	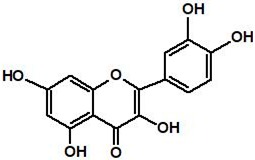	No exact mechanism associated with HSP27.
J2 (Cross linker)	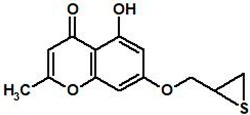	Forms a covalent bond between the cysteine thiol group of HSP27 [63].
